# Global Update and Trends of Hidden Hunger, 1995-2011: The Hidden Hunger Index

**DOI:** 10.1371/journal.pone.0143497

**Published:** 2015-12-16

**Authors:** Julie C. Ruel-Bergeron, Gretchen A. Stevens, Jonathan D. Sugimoto, Franz F. Roos, Majid Ezzati, Robert E. Black, Klaus Kraemer

**Affiliations:** 1 Department of International Health, Johns Hopkins Bloomberg School of Public Health, Baltimore, Maryland, United States of America; 2 Department of Health Statistics and Information Services, The World Health Organization, Geneva, Switzerland; 3 Vaccine and Infectious Disease Division, Fred Hutchinson Cancer Research Center, Seattle, Washington, United States of America; 4 DSM Nutritional Products, Kaiseraugst, Switzerland; 5 Environmental and Global Health Research Group, Imperial College, London, United Kingdom; 6 *Sight and Life*, Kaiseraugst, Switzerland; Oklahoma State University, UNITED STATES

## Abstract

**Background:**

Deficiencies in essential vitamins and minerals–also termed hidden hunger–are pervasive and hold negative consequences for the cognitive and physical development of children.

**Methods:**

This analysis evaluates the change in hidden hunger over time in the form of one composite indicator–the Hidden Hunger Index (HHI)–using an unweighted average of prevalence estimates from the Nutrition Impact Model Study for anemia due to iron deficiency, vitamin A deficiency, and stunting (used as a proxy indicator for zinc deficiency). Net changes from 1995–2011 and population weighted regional means for various time periods are measured.

**Findings:**

Globally, hidden hunger improved (-6.7 net change in HHI) from 1995–2011. Africa was the only region to see a deterioration in hidden hunger (+1.9) over the studied time period; East Asia and the Pacific performed exceptionally well (-13.0), while other regions improved only slightly. Improvements in HHI were mostly due to reductions in zinc and vitamin A deficiencies, while anemia due to iron deficiency persisted and even increased.

**Interpretation:**

This analysis is critical for informing and tracking the impact of policy and programmatic efforts to reduce micronutrient deficiencies, to advance the global nutrition agenda, and to achieve the Millennium Development Goals (MDGs). However, there remains an unmet need to invest in gathering frequent, nationally representative, high-quality micronutrient data as we renew our efforts to scale up nutrition, and as we enter the post-2015 development agenda.

**Funding:**

Preparation of this manuscript was funded by *Sight and Life*. There was no funding involved in the study design, data collection, analysis, or decision to publish.

## Introduction

The burden of micronutrient malnutrition, and in particular deficiencies in key micronutrients such as iron, vitamin A, iodine, and zinc, is estimated to affect 2 billion people worldwide [1]. Pregnant women and young children who are undergoing rapid growth and development are the most vulnerable to micronutrient deficiencies and, consequently, suffer the greatest adverse effects [2]. The term *hidden hunger* is synonymous with micronutrient deficiencies and accurately describes the invisible nature of the problem and the lack of overt and evident symptoms of one or more deficiencies [3].

The pervasive nature of hidden hunger holds significant individual, social, and economic consequences. The Lancet Series on Maternal and Child Nutrition (2013) [2] estimated that 90 million children <5 years (33%) and 15% of all pregnant women suffer from subclinical vitamin A deficiency (based on a threshold of serum retinol <0.70 μmol/L), the consequences of which lead to lowered immunity and increased risk of mortality in children. Iron deficiency anemia is among the most widespread nutritional deficiencies in the world, indiscriminately affecting 1.62 billion people of all life stages, countries, and genders, and holding severe implications for the cognitive and physical development of children that begins in utero [4,5]. These effects thus hold the most severe consequences for mothers and young children, of whom nearly 20% are affected [4]. Zinc has been strongly linked with linear growth [6] and immunity [2]; although zinc status is difficult to measure accurately [7], 17% of the world’s population is at risk of zinc deficiency as measured by the availability of zinc in the national diet [2].

In low- and middle-income countries (LMIC), where the diets of nutritionally vulnerable groups continue to be inadequate, the co-occurrence of deficiencies from more than one micronutrient is common [3]. In 2013, the first global Hidden Hunger Index (HHI) was developed, with the dual objective of documenting the distribution and prevalence of three common micronutrient deficiencies (zinc, iron-deficiency anemia, and vitamin A) using a composite indicator, and of providing an advocacy tool to stimulate greater investments and attention from policy- and decision-makers to eliminate hidden hunger in high-burden countries [3]. Maintaining the same methodology used in the first HHI paper [3], the analysis presented here aims to complement and extend the information on hidden hunger provided earlier, using newly available data on vitamin A status [8] and iron deficiency anemia [9]. Additional analyses also document global and country-specific trends in hidden hunger using data over 16 years (1995–2011) in 138 countries, while comparing them with other available indices, namely the Human Development Index (HDI) [10], the Global Hunger Index (GHI) [11], and the Hunger and Nutrition Commitment Index (HANCI) [12] and their time trends, data permitting. Such comparisons offer insights on how countries have performed on some indicators versus others, and how these relate to changes in hidden hunger over time.

In line with the goals of the first paper, this update will enable public health practitioners to visualize and compare the progress or deterioration of countries with regards to their HHI score over time, and to use these trends to prioritize micronutrient interventions in their respective settings, and as part of global efforts to scale up nutrition.

## Methodology

### Sources of data

Estimates of the prevalence of low serum retinol (< 0.70 μmol/L), iron-amenable anemia (Hemoglobin (Hb) < 110 g/L), and the prevalence of stunting (height-for-age Z score (HAZ) <-2, using the 2006 World Health Organization (WHO)’s growth standards [13]) among children <5 years were accessed for 138 countries from 1995–2011. Complete details on data sources and statistical methods were published elsewhere [8,9,14]. Briefly, population-representative data that measured each indicator were used. For anemia and serum retinol, data from the WHO Vitamin and Mineral Nutrition Information System (VMNIS) database [15], data provided or reported by other national and international agencies, and anonymized individual measurements of Hb in health examination surveys such as the Demographic and Health Surveys (DHS) were accessed [9,16]. For stunting, summary statistics were accessed from the WHO’s Global Database on Child Growth and Malnutrition [17], and from anonymized individual anthropometric measurements from nationally or regionally representative household surveys, and summary statistics from preliminary reports not yet included in the WHO’s database [14].

The full distribution of child HAZ and Hb for each country and year was estimated using Bayesian hierarchical mixture models. Trends were modeled over time as a linear trend plus a smooth non-linear trend. The prevalence of low serum retinol was estimated by extending the model used for child anthropometry to a Bayesian hierarchical probit model, which included linear time trends and time-varying covariates. The prevalence of anemia that would be eliminated if dietary iron were sufficient was calculated based on the observed mean increase in hemoglobin of anemic children who received iron supplementations, using data from a recent meta-analysis (8 g/L [95% CI 5–11]) [18]. To model the counterfactual scenario of adequate dietary iron, this shift was applied to all children that fell below the cutoff for total anemia. The difference between the estimated prevalence of anemia and the counterfactual prevalence of anemia was considered the iron-amenable anemia prevalence.

### Development of the Hidden Hunger Index

The construction of the 2015 HHI mirrors that which was used for the 2013 HHI, using indicators of iron-amenable anemia, vitamin A, and zinc. Note that zinc deficiency estimates are based on the prevalence of stunting among children 0–5 years, as recommended by the International Zinc Nutrition Consultative Group (IZiNCG) [19]. As opposed to the 2013 HHI publication, which represented a snapshot in time, the 2015 HHI update instead estimates an HHI score for each year over the time period of 1995–2011. (HHI scores for each country and year are provided in S1 Table.) The selection of these three micronutrients was based on the known and significant burden of these deficiencies in pre-school children, as well as on the availability of time trend data for each country and micronutrient from 1995–2011 among children under five. As stated above, the country-level annual HHI scores represent a composite index of a mixture of survey and model-based estimates of the prevalence of each micronutrient deficiency. The three micronutrients are equally weighted in the calculation of the HHI, where the HHI score = [iron deficiency-amenable anemia (%) + vitamin A deficiency (%) + stunting (%)] / 3. The HHI score is rescaled to range from 0 (best) to 100 (worst).

### Statistical Analysis

The change in HHI score between 1995 and 2011 was calculated for each country by subtracting the 1995 score from the 2011 score. This represented a “net” change, with possible change in scores ranging from negative to positive values indicating reductions and increases in the prevalence of hidden hunger among under-5 year-olds, respectively. The HHI score was categorized by the severity of the prevalence of hidden hunger, with scores between 0 and 14.9 considered mild, 15.0–24.9 considered moderate, 25–39.9 severe, and 40–100 alarmingly high. Although these severity categories are not necessarily associated with the risk of micronutrient deficiency associated morbidity, they provide for ease of interpretation and comparability between countries and over time. Regional and sub-regional HHI estimates were calculated to capture trends in hidden hunger over time, weighted by the average population under 5 years of age for the period 1995–2010 [20]. Regions were defined based on the UNICEF categorization of countries; sub-regions represent pertinent geographic delineations within regions with, for instance, West Africa and East and Southern Africa within Sub-Saharan Africa. The stratification of results by sub-region highlights the hidden hunger patterns that exist within each of the larger regions. Estimates of the standard deviation at a region, sub-region, or global level do not capture within-country heterogeneity in the HHI score. Pearson correlations were used to make comparisons with other indices, such as the HDI [10] and the GHI [11], both over time as well as for the most recent years available. A qualitative comparison of the HHI and the HANCI [21] was also performed; statistical analysis with this index was not possible, given data availability for only 45 countries, as well as the nature of the HANCI, which provides countries with a ranking based on various legal, policy, and budgetary themes rather than a score *per se*. Thus, the qualitative comparison involved a comparison of high performing countries under the HANCI and their performance on the HHI. For example, did the top-ranking HANCI country have mild or moderate HHI? Similarly, did countries which performed poorly on the HANCI have severe and/or alarmingly high HHI? Statistical analyses were conducted using STATA v13x (Stata Corp, College Station, TX).

## Results

**Fig 1:**
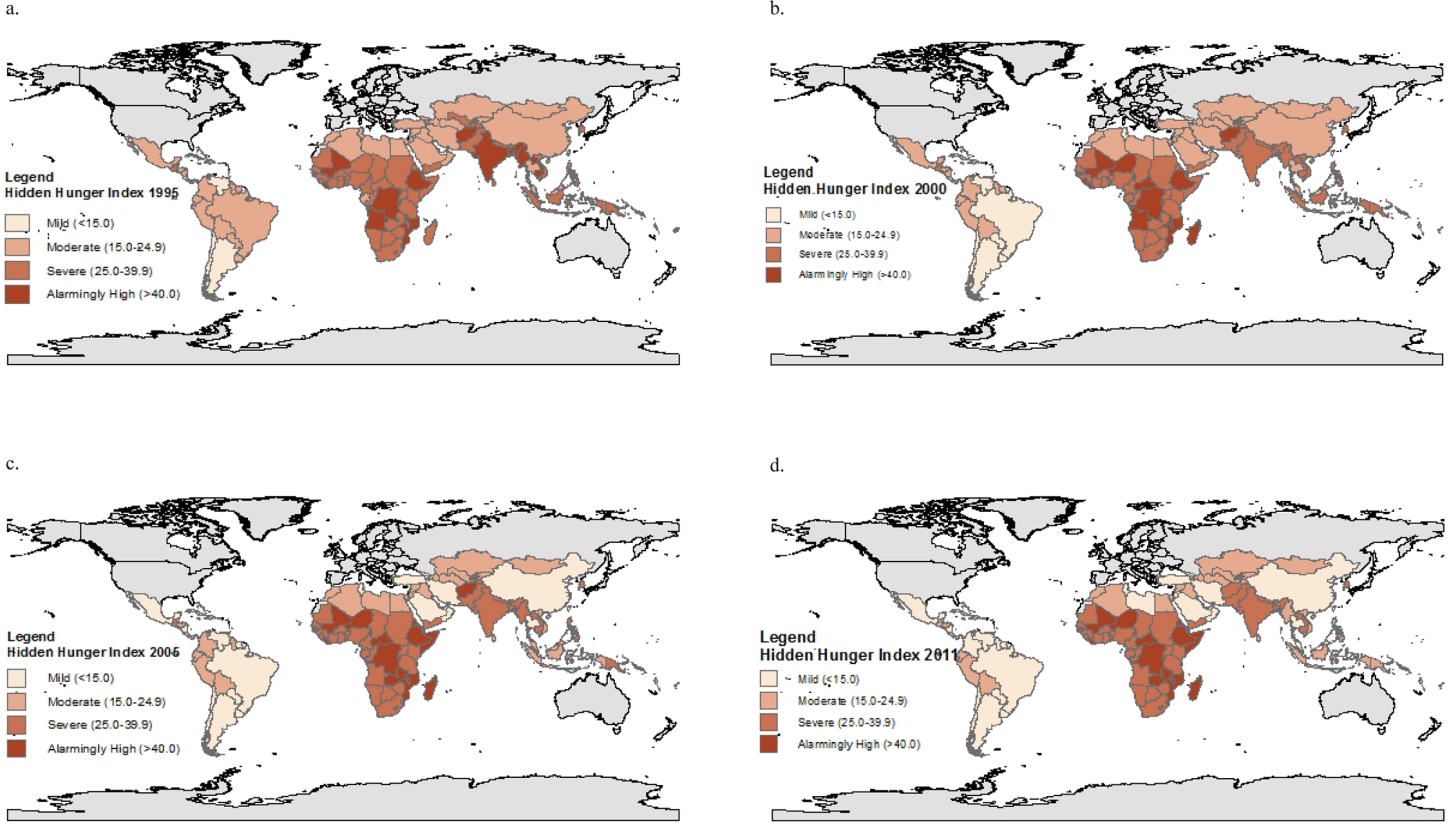
a: Global map presenting HHI scores in 1995. b: Global map presenting HHI scores in 2000. c: Global map presenting HHI scores in 2005. d: Global map presenting HHI scores in 2011.

**Fig 2:**
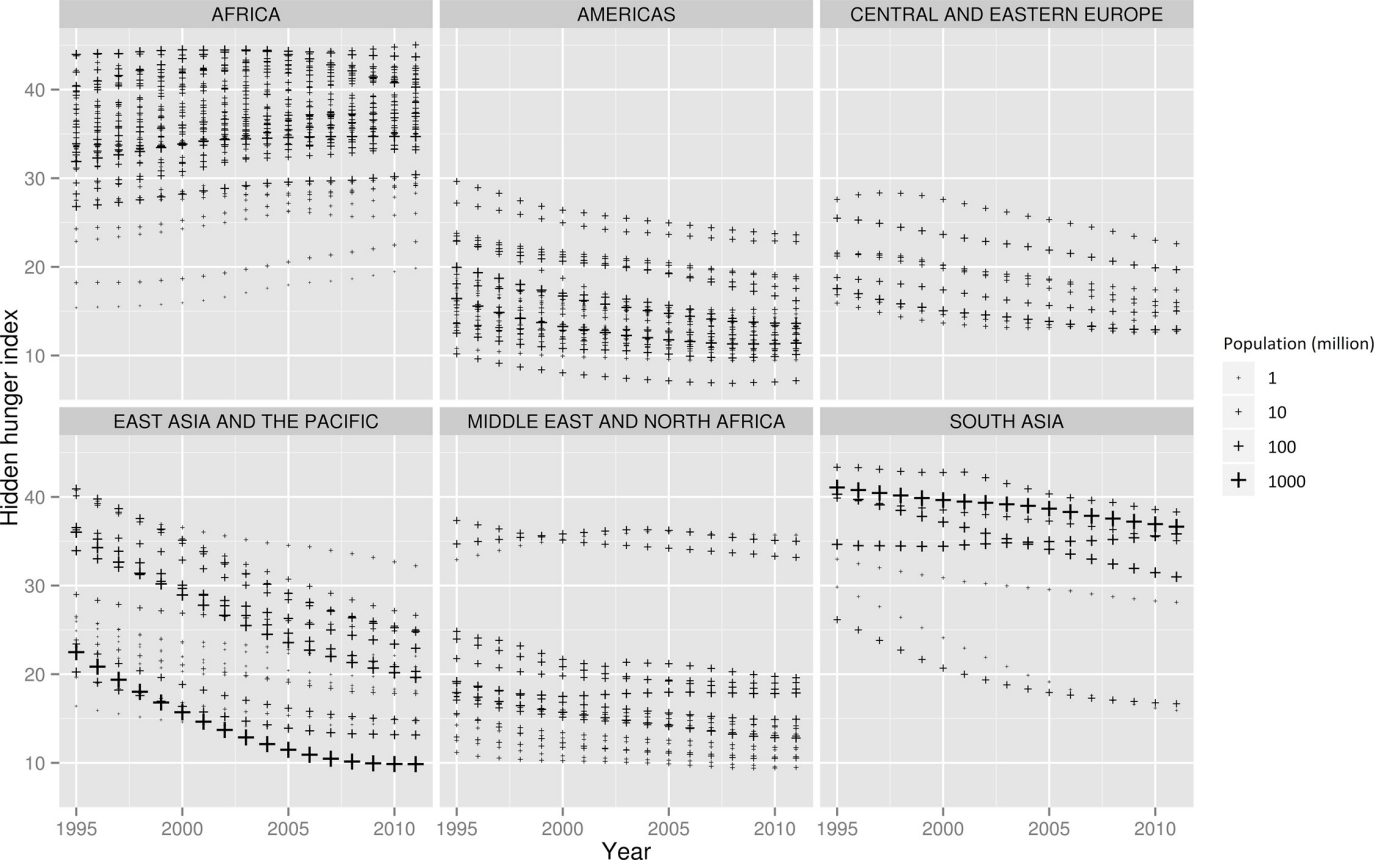
Hidden Hunger Index Score 1995–2011, by Region and Country.

The weighted global average net change (±standard deviation of the net change) in HHI (1995 to 2011) was a decrease of 6.7±5.7 (Table 1). Table 1 and Fig 1A–1D summarize regional and country trends in HHI in 5-year increments, and show that a total of 38 countries (27.5%) had an increase and 100 countries (72.5%) a reduction in HHI over the 16-year period (Table 1). Africa was the only region to experience an overall increase in hidden hunger from 1995 to 2011 (Fig 2), with a mean increase of 1.9±3.2; this increase was higher in West and Central Africa (2.7±2.3) as compared to East and Southern Africa (1.1±3.6) (Table 1). All other regions made progress in reducing hidden hunger, with East Asia and the Pacific being the top performing region (-13.0±2.0) and the remaining regions achieving only modest reductions (ranging from -3.4 to -4.8) (Table 1). The difference between the first and last years (1995 vs 2011) is striking, in that few countries in Africa had an HHI>40 in 1995 (Fig 1A). In 2011, however, almost a dozen African countries had reached this level, with no remaining Asian countries in this category (Fig 1D).

**Table 1:** Summary Statistics of Net Change and Mean Hidden Hunger Index (HHI) Score 1995–2011, by UNICEF Region and Geographical Subregion.

Region	n	Net HHI change 1995–2011	HHI All Years (1995–2011)	HHI 1995	HHI 2000	HHI 2005	HHI 2011	countries with increase	countries with decrease
		Mean (SD)	Mean (SD)	Mean (SD)	Mean (SD)	Mean (SD)	Mean (SD)	n (%)	n (%)
Africa	46	1.90 (3.21)	36.97 (4.75)	35.43 (5.18)	36.85 (5.07)	37.61 (4.66)	37.33 (3.94)	35 (76.1)	11 (23.9)
West and Central Africa	24	2.74 (2.37)	36.52 (4.13)	34.58 (4.10)	36.34 (4.27)	37.13 (4.15)	37.32 (3.89)	19 (79.2)	5 (20.8)
East and Southern Africa	22	1.06 (3.74)	37.42 (5.25)	36.29 (6.05)	37.36 (5.82)	38.08 (5.17)	37.34 (4.07)	16 (72.7)	6 (27.3)
Americas	33	-4.83 (1.59)	14.67 (3.85)	17.76 (3.91)	15.24 (3.75)	13.85 (3.56)	12.93 (3.04)	0 (0)	33 (100)
Central America	8	-6.07 (1.41)	16.88 (3.65)	20.71 (3.43)	17.63 (3.24)	15.78 (3.33)	14.65 (3.22)	0 (0)	8 (100)
South America	11	-4.48 (1.46)	13.73 (3.33)	16.61 (3.34)	14.25 (3.41)	13.00 (3.25)	12.13 (2.42)	0 (0)	11 (100)
Caribbean	14	-3.41 (1.04)	15.07 (5.34)	17.33 (5.75)	15.38 (5.54)	14.49 (5.29)	13.92 (5.15)	0 (0)	14 (100)
Middle East and North Africa	20	-3.44 (1.95)	19.51 (6.98)	21.67 (6.45)	19.77 (7.01)	19.11 (7.30)	18.23 (7.25)	2 (10)	18 (90)
Middle East	14	-4.37 (1.00)	18.22 (6.50)	20.87 (6.68)	18.67 (6.49)	17.68 (6.62)	16.49 (6.67)	0 (0)	14 (100)
North Africa	6	-2.47 (2.34)	20.85 (7.22)	22.51 (6.60)	20.92 (7.88)	20.60 (8.24)	20.03 (7.95)	2 (33.3)	4 (66.7)
Central and Eastern Europe	9	-4.74 (0.92)	17.33 (3.97)	20.06 (3.71)	18.10 (4.22)	16.62 (3.89)	15.32 (3.21)	0 (0)	9 (100)
South Asia	8	-4.40 (2.61)	37.94 (3.08)	40.14 (2.77)	38.63 (2.93)	37.57 (3.07)	35.75 (3.01)	1 (12.5)	7 (87.5)
East Asia and the Pacific	21	-12.97 (2.09)	17.69 (7.33)	25.97 (6.35)	19.51 (6.60)	15.17 (6.24)	13.00 (5.27)	0 (0)	21 (100)
Global	138	-6.72 (5.73)	25.90 (11.53)	29.96 (9.40)	26.86 (10.98)	24.81 (12.08)	23.25 (12.07)	38 (27.5)	100 (72.5)

SD, standard deviation of the country-level net change in HHI or mean HHI across the countries in each region or sub-region; Note: All regional and sub-regional summary statistics are weighted by country size, calculated as mean country population in 1995 and 2010, as presented by the United Nations Population Division 2014.

**Fig 3:**
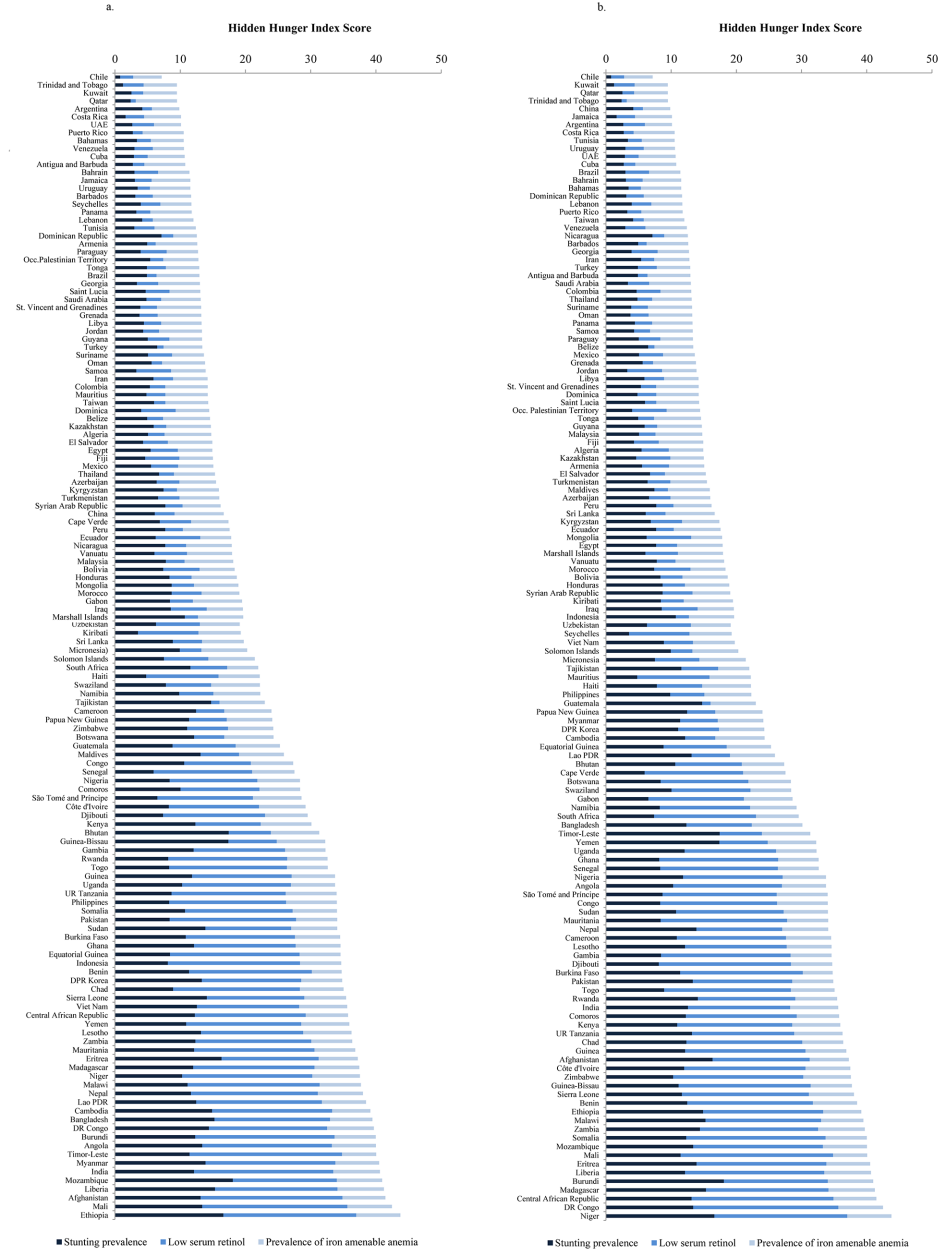
a: Hidden Hunger Index in 1995, by micronutrient deficiency. b: Hidden Hunger Index in 2011, by micronutrient deficiency.

**Fig 4:**
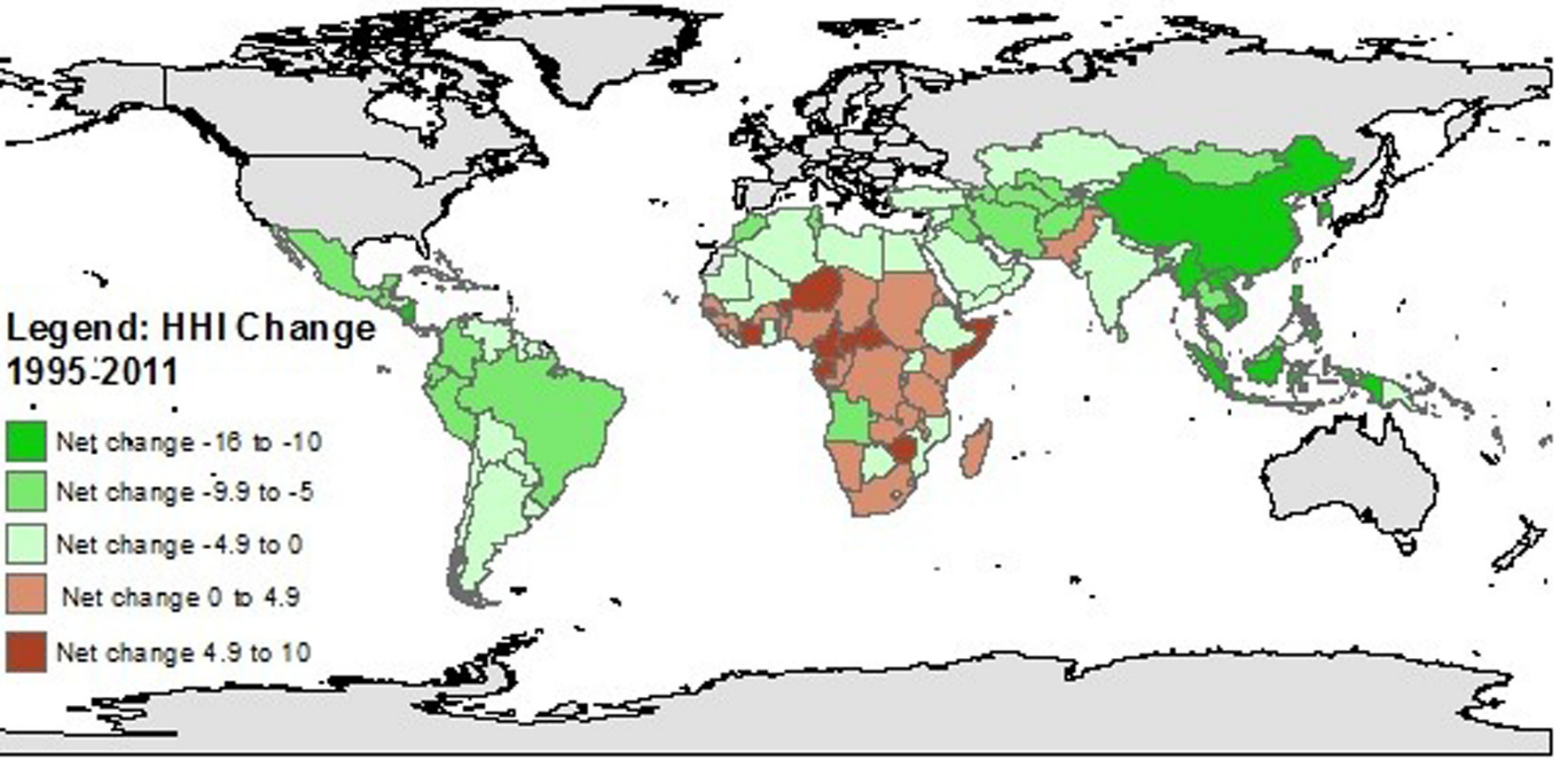
Global map presenting net change in hidden hunger index (HHI) scores, 1995–2011.

A ranking of countries from the lowest to highest HHI score in 1995 and 2011 respectively (Fig 3A and 3B), shows that Chile had the lowest score of all 138 countries to be evaluated in both years (10.2 and 7.2 in 1995 and 2011 respectively) and that Ethiopia had the highest score in 1995 (44.0), and Niger in 2011 (45.0). All but one of the 20 countries with the highest HHI score in 2011 were in Africa (Afghanistan being the exception); the 20 countries with the lowest HHI in 2011 were from the Americas, the Middle East, and North Africa (MENA), and the East Asia and the Pacific Region (EAP) (Fig 3B). This picture is quite different to that in 1995, where the 20 countries with the highest HHI were distributed more equally across four regions (EAP, South Asia, MENA, and Africa) (Fig 3A). Fig 4 illustrates the magnitude of change in HHI between 1995 and 2011, where most of the worst performers were African countries, whereas the top performers were largely from East and South Asia. A heat map of all of the countries included and their HHI scores for each year from 1995–2011 is available in S1 Table.

Fig 3A and 3B also demonstrate that the contribution of each micronutrient deficiency to the score varies based on the severity of hidden hunger. In countries with severe hidden hunger (HHI>25), zinc deficiency (as proxied by stunting) and vitamin A deficiency generally contribute a larger proportion to the HHI score than iron-amenable anemia. As countries improve their hidden hunger, however, anemia due to iron deficiency accounts for a greater–often increasing–proportion of the HHI than zinc or vitamin A deficiencies. This pattern demonstrates that much of the progress in the reduction of hidden hunger over time is attributable to reductions in zinc (proxied by stunting) and vitamin A deficiency rather than in anemia. An example of this phenomenon is found in Bangladesh, where the prevalence of zinc deficiency (as proxied by stunting), vitamin A deficiency, and iron-amenable anemia was 65%, 37%, and 19% in 1995, respectively, and 38%, 31%, and 24% in 2011, respectively. This represents a 40% reduction in the prevalence of zinc deficiency, and a 16% reduction in the prevalence of vitamin A deficiency, concurrent with a 26% increase in the prevalence of iron-amenable anemia. Similarly, in countries such as Chile–a country whose HHI is low throughout the 16-year time period–reductions in the prevalence of zinc deficiency and vitamin A deficiency (4% and 14% in 1995, respectively, and 2% and 6% in 2011, respectively) are mirrored by an increase in the prevalence of iron-amenable anemia (12.5% and 13% in 1995 and 2011, respectively). In the interpretation of these results of the relative contribution of micronutrient deficiencies to the overall HHI score, it is important to note that, despite observed reductions in stunting prevalence, zinc deficiency accounts for a small proportion of stunting. Stunting prevalence may therefore be reduced due to other factors, while zinc deficiency could remain unchanged, or even worsen [2].

**Fig 5:**
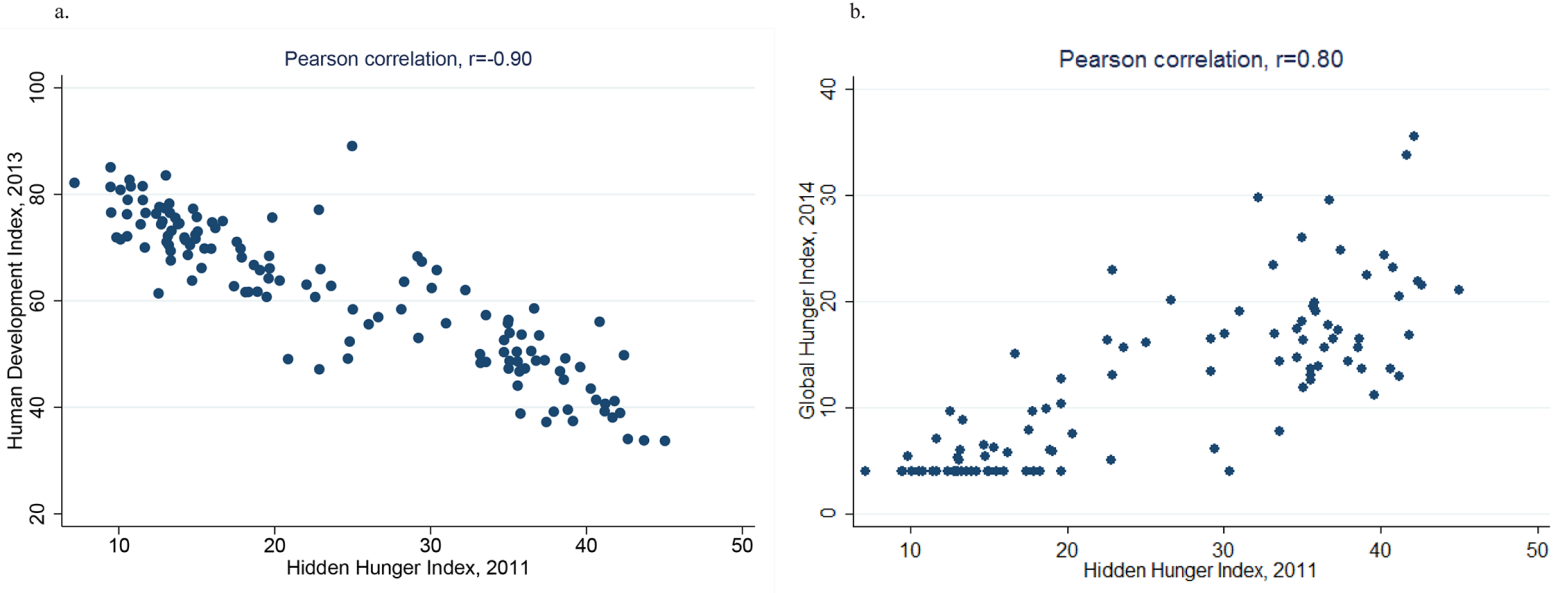
a: Correlation between the 2013 Human Development Index and the 2011 Hidden Hunger Index. b: Correlation between the 2014 Global Hunger Index and the 2011 Hidden Hunger Index.

As compared to other indices closely linked to nutrition, a statistically significant correlation was shown with the HDI 2013 (Pearson’s r = -0.90, Fig 5A) and the GHI 2014 (Pearson’s r = 0.80, Fig 5B). The correlations go in the expected directions, indicating that countries with higher human development have correspondingly lower levels of hidden hunger (Fig 5A). These associations, however, are not generalizable to the comparison of country performance over time; for example, Mozambique, whose improvement in the HDI was noteworthy, stagnated in its HHI score over the same time period. Similarly, Rwanda, Niger, and Malawi, which reduced their score on the GHI by more than 10 points in the 19-year time period (1995 to 2014) [11], saw increases in their HHI score from 1995 to 2011.

In comparing the HHI with the HANCI, the association is often contradictory (not pictured). The HANCI ranks country governments by political commitment to nutrition, based on the premise that this represents an essential component of reducing hunger and undernutrition [21]. This proves to be true for HANCI top-ranking country Guatemala, which achieved a reduction of 6 in its HHI between 1995 and 2011 [21]. However, other countries such as Myanmar, which ranked third from last in the HANCI, also achieved the third highest net reduction in hidden hunger from 1995–2011 (-16.1, Table 1).

## Discussion

This analysis provides an update on the magnitude and distribution of hidden hunger globally, and an evaluation of progress over time from 1995 to 2011, at both regional and country levels. The analysis of time trend data in this update of the HHI is critical. It allows governments and development practitioners to visualize how their country has fared over time, not only in terms of absolute numbers, but also in terms of progress or deterioration over the 16 years from 1995 to 2011 as compared to other countries in their region, or globally. The results, which mirror global nutrition and human development trends, are sobering. They are especially so for Africa, which is the only region to have experienced a rise in hidden hunger over the 16 years, and currently has the highest HHI average score, and many of the worst performing countries in terms of both severity and deterioration over time. The consequences of such sustained and widespread hidden hunger, not only in Africa but also in the other regions, are severe, and represent long-lasting threats to each country’s social and economic development [11]. Thus, and in the context of increased attention to nutrition globally (including in Africa), large-scale micronutrient programs can use the findings of this analysis to better target their programs to vulnerable populations in the highest burden countries, and to specific micronutrients of concern.

In evaluating hidden hunger trends, the level at which each country began (1995) does appear to be a strong predictor of performance over time. Seychelles, for example, was among the 20 countries with the lowest HHI in 1995, but was also among the top 20 worst performers in terms of net change in HHI over the 16-year period studied. Potential uncertainty due to questions about the quality of data from some parts of this small island state, and/or a low starting point (*i*.*e*., a lower prevalence of hidden hunger) which leaves little room for improvement may have contributed to this result. The latter is supported by findings for some of the 20 other countries with the highest HHI in 1995, which achieved the largest reductions in hidden hunger over the 16-year time period (for example, Myanmar and Cambodia). As such, countries with severe hidden hunger (HHI>25) may be able to achieve more impressive reductions over time than countries which started with mild hidden hunger (HHI<25).

Although, overall, the HHI correlates with other global indicators of hunger and development, especially the HDI and GHI, our analyses show that there are also some disconnects and that some countries are clear outliers. The discrepancy between the indices is likely to be related to the inherent differences in the drivers of undernutrition which are quantified by each indicator. The GHI captures the multidimensional aspects and consequences of hunger and undernutrition, whereas the HHI reflects the more pervasive and underlying nature of micronutrient deficiencies, the burden and consequences of which are more difficult to isolate [3]. Similarly, the HANCI focuses strongly on the tenants of legal, political, and financial commitment. It emphasizes that a country’s ranking thus does not necessarily equate to a direct impact on nutrition outcomes, much less hidden hunger outcomes [21], which we have observed for some countries here. In fact, such contradictions between hunger indices stress the complex nature of hidden hunger and that, although economic development and political commitment inevitably play a role in improving nutrition, they are not the only factors leading to change [22]. Social policies that are equitable and pro-poor, and are matched with financial and human resource investments in health and education [22], are necessary drivers of change for improving both human development and reducing hidden hunger.

In attempting to explain the trends in hidden hunger over time in the best and worst performing countries, it is worth noting that the five worst performing countries are in Sub-Saharan Africa, and have undergone some combination of periods of significant conflict and vulnerability to food insecurity due to climate-related shocks such as drought and floods [23]. In Zimbabwe, for example, the prevalence of stunting and low serum retinol increased slightly over the 16-year time period, while iron-amenable anemia almost doubled (from 13% to 23%), with increases of approximately one percentage point per year from 1995–2005, coinciding with a period of widespread unrest in the country [24].

The remarkable progress made by some Asian countries–specifically Indonesia, Viet Nam, Cambodia, and Myanmar–is paralleled by significant economic growth. Myanmar ranked second overall in terms of average annual growth in gross national income per capita from 1990–2012, while Viet Nam ranked third in terms of income growth, and performed among the top 20 in terms of HDI improvement from 1990–2013 [10]. In addition, Viet Nam’s ranking at number 15 in the HANCI is supported by numerous nutrition and socio-economic strategies and action plans which were implemented as early as the mid-1990s, as well as its successful achievement of several MDGs, including MDG1 on poverty and hunger reduction [25,26]. The timeline under which Viet Nam achieved success in reducing hidden hunger may be representative of a time lag between political commitment and the achievement of results in nutrition, wherein the implementation of programs and policies may require years–even decades–to translate into results.

There are limitations of this paper that deserve mention. First, the HHI is limited in its scope as it does not adequately capture the extent and severity of hidden hunger in each of the analyzed countries, or for more than the three target micronutrients. Essential micronutrients such as folic acid, other B-vitamins, calcium, and others, do not have nationally representative data, and are thus not included in this index. Secondly, there was concern about the degree to which equal weighting of each micronutrient would influence each country’s HHI. Specifically, variations in relative levels of uncertainty were thought to potentially lead to biased estimates of the temporal trend in HHI. Analyses to explore this limitation indicated evidence of a slight bias in the estimated change in HHI, but this bias was not substantial enough to change our primary conclusions about regional trends (results not shown). Thus, we elected to continue with the unweighted index. Another inherent limitation of this study is the use of modeled estimates of the three indicators of interest for years in which survey data is not available. Nationally representative nutrition surveys–especially those that collect biospecimens that are used to assess the prevalence of micronutrient deficiencies–are scarce, given the financial and logistical constraints associated with collecting such data, particularly in resource-poor settings. Nevertheless, this analysis highlights the dearth of data available to measure and track micronutrient status globally, and makes a plea for increased investments in, and maintenance of, the World Health Organization’s Vitamin and Mineral Nutrition Information System (VMNIS) [15].

Lastly, we recognize the limitation related to the use of stunting prevalence as a proxy for zinc deficiency, which does not reflect the true prevalence of zinc deficiency, given that multiple factors besides zinc deficiency could impair linear growth [27]. Nevertheless, zinc continues to be the only micronutrient that has been shown to enhance linear growth, albeit with a small effect on stunting prevalence [28]. The use of data on probability of inadequate zinc intake based on zinc availability in the food supply was considered for use in this index but was deemed inadequate since it refers to national populations, and is not specific to children. Moreover, it is intended to serve as an indication of the high or low risk of inadequate zinc intake, rather than deficiency itself. Still, we conducted exploratory analysis replacing stunting with the probability of inadequate zinc intake as a proxy for zinc deficiency in the HHI, and found that the resulting HHI systematically under-estimated, although only by negligible proportions, the estimates derived using stunting as a proxy indicator (S2 Table). Thus, the decision to proceed with stunting as a proxy for zinc deficiency was maintained in an effort to preserve the methodology used in the 2013 HHI, as well as to use the IZiNCG recommendation [19].

## Conclusion

Deficiencies in essential vitamins and minerals–also termed hidden hunger–are pervasive, and continue to be largely hidden. The impact of hidden hunger holds significant and immediate negative consequences for the cognitive and physical development of children, as well as longer-lasting effects on productivity and economic potential in later adulthood. This time trend analysis of the hidden hunger index, a composite measure of three key micronutrient deficiencies, contributes to our global understanding of how hidden hunger has evolved over time both within individual countries and at the regional level, and how these trends compare to a number of other global indices presenting various aspects of hunger and nutrition. Both generate noteworthy results that can be used for evidence-informed decision and policy-making in the context of a growing interest in nutrition, and a parallel paucity of data on hidden hunger.

In parallel, this analysis highlights an urgent need to invest in the updating of the VMNIS, so that it continues to gather frequent, nationally representative, high-quality micronutrient data for a large number of countries and for a wider range of micronutrients. Such data is critical to inform the policy and programmatic efforts needed to advance the global nutrition agenda, and to achieve MDGs 1, 4, and 5. Looking ahead, as we enter the post-2015 development agenda period and renew our efforts to scale-up nutrition, such a global micronutrient information system is critically important to provide the urgently needed data for monitoring and tracking progress in achieving the new sustainable development goals (SDGs).

## Supporting Information

S1 Table Hidden Hunger Index by Country and Year, 1995–2011. (DOCX)

S2 Table Comparison of the Hidden Hunger Index (HHI) estimates for 2009 (unweighted) using dietary zinc data as compared to using stunting prevalence as an estimate of the prevalence of zinc deficiency. (DOCX)
