# Supplementary material for: Global Update and Trends of Hidden Hunger, 1995-2011: The Hidden Hunger Index
Source: PLoS One. 2015 Dec 16;10(12):e0143497. doi: 10.1371/journal.pone.0143497 (PMC4684416; doi:10.1371/journal.pone.0143497)
Supplement: S1 Table — (DOCX) [file pone.0143497.s001.docx]

**S1 Table: Hidden Hunger Index by Country and Year, 1995-2011**

|  | **Year** | | | | | | | | | | | | | | | | |
| --- | --- | --- | --- | --- | --- | --- | --- | --- | --- | --- | --- | --- | --- | --- | --- | --- | --- |
| **Region/Sub-region/Country** | **1995** | **1996** | **1997** | **1998** | **1999** | **2000** | **2001** | **2002** | **2003** | **2004** | **2005** | **2006** | **2007** | **2008** | **2009** | **2010** | **2011** |
| **Africa** |  |  |  |  |  |  |  |  |  |  |  |  |  |  |  |  |  |
| **East and Southern Africa** |  |  |  |  |  |  |  |  |  |  |  |  |  |  |  |  |  |
| Seychelles | 15 | 16 | 16 | 16 | 16 | 16 | 16 | 17 | 17 | 18 | 18 | 18 | 18 | 19 | 19 | 20 | 20 |
| Mauritius | 18 | 18 | 18 | 18 | 19 | 19 | 19 | 19 | 20 | 20 | 21 | 21 | 21 | 22 | 22 | 23 | 23 |
| South Africa | 27 | 27 | 27 | 28 | 28 | 28 | 29 | 29 | 29 | 29 | 30 | 30 | 30 | 30 | 30 | 30 | 30 |
| Namibia | 28 | 28 | 28 | 28 | 28 | 28 | 29 | 29 | 29 | 29 | 29 | 29 | 30 | 30 | 30 | 30 | 30 |
| Swaziland | 28 | 28 | 28 | 28 | 28 | 28 | 28 | 28 | 28 | 28 | 28 | 28 | 29 | 29 | 29 | 29 | 29 |
| Zimbabwe | 30 | 30 | 30 | 30 | 31 | 32 | 33 | 34 | 35 | 36 | 37 | 37 | 38 | 38 | 38 | 39 | 39 |
| Botswana | 30 | 30 | 29 | 29 | 29 | 29 | 28 | 28 | 28 | 28 | 28 | 28 | 28 | 29 | 29 | 29 | 29 |
| Comoros | 32 | 33 | 33 | 33 | 34 | 34 | 34 | 35 | 35 | 35 | 36 | 36 | 36 | 36 | 36 | 37 | 37 |
| Kenya | 33 | 33 | 33 | 33 | 34 | 34 | 34 | 34 | 35 | 35 | 36 | 36 | 36 | 36 | 37 | 37 | 37 |
| Rwanda | 34 | 34 | 34 | 34 | 35 | 35 | 36 | 36 | 36 | 37 | 37 | 37 | 37 | 37 | 37 | 37 | 37 |
| UR Tanzania | 34 | 34 | 35 | 35 | 36 | 36 | 36 | 37 | 37 | 37 | 37 | 37 | 37 | 37 | 37 | 37 | 37 |
| Uganda | 34 | 34 | 34 | 34 | 34 | 34 | 34 | 34 | 34 | 34 | 34 | 34 | 34 | 34 | 33 | 33 | 33 |
| Somalia | 35 | 35 | 36 | 36 | 37 | 37 | 38 | 39 | 39 | 39 | 40 | 40 | 41 | 41 | 41 | 41 | 41 |
| Lesotho | 38 | 38 | 37 | 37 | 37 | 37 | 37 | 37 | 37 | 37 | 36 | 36 | 36 | 36 | 36 | 36 | 36 |
| Zambia | 38 | 38 | 39 | 39 | 39 | 40 | 40 | 40 | 40 | 41 | 41 | 41 | 41 | 41 | 41 | 41 | 41 |
| Eritrea | 39 | 39 | 38 | 38 | 38 | 38 | 39 | 39 | 39 | 40 | 40 | 40 | 41 | 41 | 41 | 42 | 42 |
| Madagascar | 39 | 40 | 41 | 41 | 42 | 42 | 42 | 43 | 43 | 43 | 43 | 43 | 43 | 43 | 43 | 43 | 42 |
| Malawi | 40 | 40 | 40 | 41 | 41 | 41 | 42 | 42 | 42 | 42 | 42 | 42 | 42 | 42 | 41 | 41 | 41 |
| Burundi | 40 | 41 | 42 | 42 | 42 | 42 | 42 | 42 | 43 | 43 | 43 | 43 | 43 | 43 | 42 | 42 | 42 |
| Angola | 41 | 41 | 41 | 40 | 40 | 40 | 40 | 40 | 40 | 40 | 39 | 38 | 37 | 36 | 36 | 35 | 35 |
| Mozambique | 42 | 42 | 42 | 42 | 42 | 42 | 42 | 42 | 42 | 42 | 42 | 42 | 42 | 41 | 41 | 41 | 41 |
| Ethiopia | 44 | 44 | 44 | 44 | 44 | 45 | 45 | 45 | 45 | 44 | 44 | 44 | 43 | 42 | 41 | 41 | 40 |
| **West and Central Africa** |  |  |  |  |  |  |  |  |  |  |  |  |  |  |  |  |  |
| Cape Verde | 23 | 23 | 23 | 24 | 24 | 24 | 25 | 25 | 25 | 26 | 26 | 27 | 27 | 27 | 28 | 28 | 28 |
| Gabon | 24 | 24 | 24 | 25 | 25 | 25 | 26 | 26 | 26 | 27 | 27 | 28 | 28 | 28 | 29 | 29 | 29 |
| Cameroon | 28 | 29 | 29 | 30 | 30 | 31 | 31 | 32 | 32 | 33 | 33 | 34 | 34 | 34 | 35 | 35 | 36 |
| Congo | 31 | 31 | 32 | 32 | 32 | 32 | 33 | 33 | 33 | 34 | 34 | 34 | 35 | 35 | 35 | 35 | 35 |
| Senegal | 31 | 31 | 32 | 32 | 32 | 32 | 32 | 32 | 32 | 32 | 32 | 33 | 33 | 33 | 33 | 33 | 34 |
| Nigeria | 32 | 32 | 33 | 33 | 34 | 34 | 34 | 34 | 34 | 35 | 35 | 35 | 35 | 35 | 35 | 35 | 35 |
| São Tomé and Príncipe | 32 | 33 | 33 | 33 | 34 | 34 | 34 | 34 | 35 | 35 | 35 | 35 | 35 | 35 | 35 | 35 | 35 |
| Côte d'Ivoire | 33 | 33 | 33 | 33 | 34 | 34 | 34 | 35 | 36 | 36 | 37 | 37 | 37 | 38 | 38 | 38 | 39 |
| Guinea-Bissau | 33 | 34 | 34 | 35 | 35 | 36 | 37 | 37 | 38 | 38 | 38 | 38 | 38 | 39 | 39 | 39 | 39 |
| Gambia | 33 | 34 | 34 | 34 | 34 | 34 | 34 | 35 | 35 | 35 | 35 | 35 | 35 | 35 | 35 | 35 | 36 |
| Togo | 34 | 34 | 34 | 34 | 34 | 34 | 35 | 35 | 35 | 35 | 35 | 36 | 36 | 36 | 36 | 36 | 36 |
| Guinea | 34 | 34 | 34 | 34 | 35 | 35 | 35 | 36 | 36 | 36 | 37 | 37 | 37 | 37 | 38 | 38 | 38 |
| Burkina Faso | 35 | 35 | 36 | 36 | 36 | 36 | 36 | 36 | 36 | 36 | 36 | 36 | 36 | 36 | 36 | 36 | 36 |
| Ghana | 36 | 36 | 36 | 36 | 36 | 36 | 36 | 35 | 35 | 35 | 35 | 35 | 34 | 34 | 34 | 34 | 34 |
| Equatorial Guinea | 36 | 35 | 34 | 33 | 31 | 30 | 29 | 28 | 28 | 27 | 26 | 26 | 26 | 26 | 26 | 26 | 26 |
| Benin | 36 | 36 | 37 | 37 | 37 | 37 | 37 | 38 | 38 | 38 | 38 | 39 | 39 | 39 | 39 | 39 | 40 |
| Chad | 36 | 37 | 37 | 37 | 38 | 38 | 38 | 38 | 38 | 38 | 37 | 37 | 37 | 37 | 37 | 37 | 37 |
| Sierra Leone | 37 | 37 | 38 | 39 | 40 | 41 | 41 | 40 | 40 | 40 | 40 | 40 | 40 | 39 | 39 | 39 | 39 |
| Central African Republic | 37 | 38 | 39 | 39 | 40 | 40 | 41 | 41 | 42 | 42 | 42 | 43 | 43 | 43 | 43 | 43 | 43 |
| Mauritania | 38 | 38 | 38 | 38 | 38 | 38 | 38 | 37 | 37 | 37 | 37 | 36 | 36 | 35 | 35 | 35 | 35 |
| Niger | 40 | 40 | 41 | 41 | 42 | 42 | 42 | 43 | 43 | 44 | 44 | 44 | 44 | 44 | 45 | 45 | 45 |
| Democratic Republic of the Congo | 40 | 41 | 42 | 42 | 43 | 44 | 44 | 44 | 44 | 44 | 44 | 44 | 44 | 44 | 44 | 44 | 44 |
| Liberia | 42 | 43 | 43 | 42 | 41 | 41 | 40 | 40 | 41 | 42 | 42 | 42 | 42 | 42 | 42 | 42 | 42 |
| Mali | 44 | 44 | 44 | 44 | 44 | 44 | 44 | 44 | 43 | 43 | 43 | 43 | 42 | 42 | 42 | 41 | 41 |
| **Central and Eastern Europe** |  |  |  |  |  |  |  |  |  |  |  |  |  |  |  |  |  |
|  |  |  |  |  |  |  |  |  |  |  |  |  |  |  |  |  |  |
| Armenia | 16 | 15 | 15 | 14 | 14 | 14 | 14 | 13 | 13 | 13 | 13 | 13 | 13 | 14 | 14 | 15 | 15 |
| Georgia | 17 | 17 | 16 | 16 | 15 | 15 | 14 | 14 | 14 | 14 | 14 | 13 | 13 | 13 | 13 | 13 | 13 |
| Turkey | 18 | 17 | 16 | 16 | 16 | 15 | 15 | 15 | 14 | 14 | 14 | 14 | 13 | 13 | 13 | 13 | 13 |
| Kazakhstan | 19 | 19 | 18 | 18 | 18 | 17 | 17 | 17 | 16 | 16 | 16 | 15 | 15 | 15 | 15 | 15 | 15 |
| Azerbaijan | 21 | 21 | 21 | 21 | 21 | 20 | 20 | 19 | 19 | 19 | 19 | 18 | 17 | 17 | 16 | 16 | 16 |
| Kyrgyzstan | 22 | 21 | 21 | 21 | 20 | 20 | 19 | 19 | 19 | 19 | 19 | 18 | 18 | 18 | 18 | 17 | 17 |
| Turkmenistan | 22 | 22 | 22 | 21 | 21 | 20 | 20 | 19 | 19 | 18 | 18 | 17 | 17 | 16 | 16 | 16 | 16 |
| Uzbekistan | 26 | 25 | 25 | 25 | 24 | 24 | 23 | 23 | 23 | 22 | 22 | 22 | 21 | 21 | 20 | 20 | 20 |
| Tajikistan | 28 | 28 | 28 | 28 | 28 | 28 | 27 | 27 | 26 | 26 | 25 | 25 | 25 | 24 | 24 | 23 | 23 |
| **East Asia and the Pacific** |  |  |  |  |  |  |  |  |  |  |  |  |  |  |  |  |  |
|  |  |  |  |  |  |  |  |  |  |  |  |  |  |  |  |  |  |
| Tonga | 16 | 16 | 16 | 15 | 15 | 15 | 14 | 14 | 14 | 14 | 14 | 14 | 14 | 14 | 14 | 15 | 15 |
| Samoa | 18 | 17 | 17 | 16 | 16 | 15 | 15 | 14 | 14 | 14 | 14 | 14 | 13 | 13 | 13 | 13 | 13 |
| Taiwan | 18 | 17 | 16 | 16 | 15 | 14 | 14 | 13 | 13 | 13 | 12 | 12 | 12 | 12 | 12 | 12 | 12 |
| Fiji | 20 | 19 | 18 | 18 | 17 | 17 | 16 | 16 | 16 | 16 | 15 | 15 | 15 | 15 | 15 | 15 | 15 |
| Thailand | 20 | 19 | 18 | 18 | 17 | 16 | 16 | 15 | 15 | 14 | 14 | 14 | 13 | 13 | 13 | 13 | 13 |
| China | 23 | 21 | 19 | 18 | 17 | 16 | 15 | 14 | 13 | 12 | 12 | 11 | 11 | 10 | 10 | 10 | 10 |
| Vanuatu | 23 | 22 | 22 | 21 | 21 | 20 | 20 | 20 | 20 | 20 | 19 | 19 | 19 | 19 | 19 | 18 | 18 |
| Malaysia | 23 | 22 | 21 | 20 | 20 | 19 | 18 | 18 | 17 | 17 | 16 | 16 | 16 | 15 | 15 | 15 | 15 |
| Mongolia | 24 | 23 | 23 | 22 | 22 | 21 | 21 | 20 | 20 | 20 | 19 | 19 | 18 | 18 | 18 | 18 | 18 |
| Marshall Islands | 25 | 24 | 24 | 23 | 23 | 22 | 21 | 21 | 20 | 20 | 19 | 19 | 19 | 18 | 18 | 18 | 18 |
| Kiribati | 26 | 25 | 24 | 23 | 23 | 22 | 22 | 21 | 21 | 21 | 20 | 20 | 20 | 20 | 20 | 20 | 20 |
| Micronesia (Federated States of) | 26 | 26 | 25 | 25 | 24 | 24 | 23 | 23 | 23 | 23 | 22 | 22 | 22 | 22 | 22 | 22 | 22 |
| Solomon Islands | 27 | 26 | 25 | 24 | 24 | 24 | 24 | 24 | 23 | 23 | 23 | 22 | 22 | 22 | 21 | 21 | 21 |
| Papua New Guinea | 29 | 28 | 28 | 28 | 27 | 27 | 27 | 27 | 26 | 26 | 26 | 26 | 26 | 25 | 25 | 25 | 25 |
| Philippines | 34 | 33 | 32 | 31 | 31 | 30 | 29 | 28 | 28 | 27 | 26 | 26 | 25 | 24 | 24 | 23 | 23 |
| Indonesia | 36 | 34 | 33 | 31 | 30 | 29 | 28 | 27 | 26 | 25 | 24 | 23 | 22 | 21 | 21 | 20 | 20 |
| Democratic People's Republic of Korea | 36 | 36 | 35 | 35 | 34 | 33 | 32 | 31 | 30 | 29 | 28 | 28 | 27 | 27 | 26 | 25 | 25 |
| Viet Nam | 37 | 35 | 34 | 33 | 31 | 30 | 29 | 28 | 27 | 26 | 25 | 24 | 23 | 22 | 21 | 21 | 20 |
| Lao People's Democratic Republic | 40 | 39 | 38 | 37 | 36 | 35 | 34 | 33 | 32 | 32 | 31 | 30 | 29 | 28 | 28 | 27 | 27 |
| Cambodia | 40 | 39 | 38 | 37 | 36 | 35 | 34 | 32 | 31 | 30 | 29 | 28 | 27 | 26 | 26 | 25 | 25 |
| Timor-Leste | 41 | 40 | 38 | 37 | 37 | 37 | 36 | 36 | 35 | 35 | 35 | 34 | 34 | 34 | 33 | 33 | 32 |
| Myanmar | 41 | 40 | 39 | 38 | 36 | 35 | 34 | 33 | 31 | 30 | 29 | 28 | 27 | 26 | 26 | 25 | 25 |
| **Middle East and North Africa** |  |  |  |  |  |  |  |  |  |  |  |  |  |  |  |  |  |
| **Middle East** |  |  |  |  |  |  |  |  |  |  |  |  |  |  |  |  |  |
| Kuwait | 11 | 11 | 11 | 10 | 10 | 10 | 10 | 10 | 10 | 10 | 10 | 10 | 10 | 10 | 9 | 9 | 10 |
| Qatar | 13 | 12 | 12 | 11 | 11 | 11 | 11 | 11 | 10 | 10 | 10 | 10 | 10 | 10 | 10 | 10 | 10 |
| United Arab Emirates | 13 | 13 | 12 | 12 | 12 | 12 | 12 | 12 | 12 | 11 | 11 | 11 | 11 | 11 | 11 | 11 | 11 |
| Bahrain | 14 | 14 | 14 | 13 | 13 | 13 | 13 | 13 | 12 | 12 | 12 | 12 | 12 | 12 | 12 | 12 | 12 |
| Lebanon | 15 | 15 | 14 | 14 | 14 | 13 | 13 | 13 | 13 | 13 | 13 | 13 | 12 | 12 | 12 | 12 | 12 |
| Occupied Palestinian Territory | 16 | 16 | 15 | 15 | 14 | 14 | 14 | 14 | 14 | 15 | 15 | 15 | 15 | 14 | 14 | 14 | 14 |
| Saudi Arabia | 17 | 17 | 16 | 16 | 16 | 15 | 15 | 15 | 15 | 14 | 14 | 14 | 14 | 13 | 13 | 13 | 13 |
| Libyan Arab Jamahiriya | 17 | 17 | 17 | 16 | 16 | 16 | 16 | 16 | 15 | 15 | 15 | 15 | 15 | 14 | 14 | 14 | 14 |
| Jordan | 18 | 17 | 17 | 16 | 16 | 16 | 16 | 16 | 16 | 16 | 15 | 15 | 15 | 15 | 14 | 14 | 14 |
| Oman | 18 | 17 | 17 | 16 | 16 | 16 | 15 | 15 | 15 | 15 | 14 | 14 | 14 | 14 | 14 | 13 | 13 |
| Iran (Islamic Republic of) | 18 | 17 | 17 | 17 | 16 | 16 | 15 | 15 | 15 | 15 | 14 | 14 | 14 | 13 | 13 | 13 | 13 |
| Syrian Arab Republic | 22 | 21 | 21 | 20 | 20 | 20 | 20 | 20 | 20 | 19 | 19 | 19 | 19 | 19 | 19 | 19 | 19 |
| Iraq | 25 | 24 | 24 | 23 | 22 | 22 | 21 | 21 | 21 | 21 | 21 | 21 | 21 | 20 | 20 | 20 | 20 |
| Yemen | 37 | 37 | 36 | 36 | 36 | 35 | 35 | 35 | 35 | 34 | 34 | 34 | 34 | 34 | 34 | 33 | 33 |
| **North Africa** |  |  |  |  |  |  |  |  |  |  |  |  |  |  |  |  |  |
| Tunisia | 16 | 15 | 14 | 13 | 13 | 12 | 12 | 12 | 11 | 11 | 11 | 11 | 11 | 11 | 11 | 11 | 11 |
| Algeria | 19 | 19 | 18 | 18 | 17 | 17 | 17 | 16 | 16 | 16 | 16 | 15 | 15 | 15 | 15 | 15 | 15 |
| Egypt | 19 | 19 | 18 | 18 | 18 | 18 | 18 | 18 | 18 | 18 | 18 | 18 | 18 | 18 | 18 | 18 | 18 |
| Morocco | 24 | 23 | 23 | 22 | 21 | 21 | 21 | 20 | 20 | 19 | 19 | 19 | 19 | 19 | 18 | 18 | 18 |
| Djibouti | 33 | 33 | 34 | 35 | 35 | 35 | 36 | 36 | 36 | 36 | 36 | 36 | 36 | 36 | 36 | 36 | 36 |
| Sudan | 35 | 35 | 35 | 35 | 36 | 36 | 36 | 36 | 36 | 36 | 36 | 36 | 36 | 35 | 35 | 35 | 35 |
| **South Asia** |  |  |  |  |  |  |  |  |  |  |  |  |  |  |  |  |  |
|  |  |  |  |  |  |  |  |  |  |  |  |  |  |  |  |  |  |
| Sri Lanka | 26 | 25 | 24 | 23 | 22 | 21 | 20 | 19 | 19 | 18 | 18 | 18 | 17 | 17 | 17 | 17 | 17 |
| Maldives | 30 | 29 | 28 | 26 | 25 | 24 | 23 | 22 | 21 | 20 | 19 | 18 | 18 | 17 | 17 | 16 | 16 |
| Bhutan | 33 | 33 | 32 | 32 | 31 | 31 | 30 | 30 | 30 | 30 | 30 | 29 | 29 | 29 | 29 | 28 | 28 |
| Pakistan | 35 | 35 | 35 | 35 | 34 | 34 | 35 | 35 | 35 | 35 | 35 | 35 | 35 | 35 | 35 | 36 | 36 |
| Nepal | 40 | 40 | 39 | 39 | 39 | 39 | 38 | 38 | 38 | 38 | 37 | 37 | 37 | 36 | 36 | 36 | 35 |
| Bangladesh | 40 | 40 | 39 | 39 | 38 | 37 | 37 | 36 | 35 | 35 | 34 | 34 | 33 | 32 | 32 | 32 | 31 |
| India | 41 | 41 | 40 | 40 | 40 | 40 | 40 | 39 | 39 | 39 | 39 | 38 | 38 | 38 | 37 | 37 | 37 |
| Afghanistan | 43 | 43 | 43 | 43 | 43 | 43 | 43 | 42 | 42 | 41 | 40 | 40 | 40 | 39 | 39 | 39 | 38 |
| **The Americas** |  |  |  |  |  |  |  |  |  |  |  |  |  |  |  |  |  |
| **Caribbean** |  |  |  |  |  |  |  |  |  |  |  |  |  |  |  |  |  |
| Trinidad and Tobago | 11 | 11 | 10 | 10 | 10 | 10 | 10 | 10 | 10 | 10 | 10 | 10 | 10 | 9 | 9 | 10 | 10 |
| Puerto Rico | 13 | 13 | 13 | 13 | 12 | 12 | 12 | 12 | 12 | 12 | 12 | 12 | 12 | 12 | 12 | 12 | 12 |
| Bahamas | 13 | 13 | 13 | 12 | 12 | 12 | 12 | 12 | 12 | 12 | 12 | 12 | 12 | 12 | 12 | 12 | 12 |
| Cuba | 14 | 13 | 13 | 12 | 12 | 12 | 12 | 12 | 11 | 11 | 11 | 11 | 11 | 11 | 11 | 11 | 11 |
| Antigua and Barbuda | 14 | 14 | 14 | 14 | 13 | 13 | 13 | 13 | 13 | 13 | 13 | 13 | 13 | 13 | 13 | 13 | 13 |
| Jamaica | 15 | 14 | 13 | 13 | 13 | 12 | 12 | 12 | 11 | 11 | 11 | 11 | 10 | 10 | 10 | 10 | 10 |
| Barbados | 15 | 15 | 15 | 14 | 14 | 14 | 14 | 13 | 13 | 13 | 13 | 13 | 13 | 13 | 13 | 13 | 13 |
| Dominican Republic | 16 | 15 | 15 | 14 | 14 | 14 | 13 | 13 | 13 | 13 | 13 | 12 | 12 | 12 | 12 | 12 | 12 |
| Saint Lucia | 17 | 17 | 17 | 16 | 16 | 16 | 16 | 16 | 15 | 15 | 15 | 15 | 15 | 15 | 14 | 14 | 14 |
| Saint Vincent and the Grenadines | 17 | 17 | 16 | 16 | 16 | 16 | 15 | 15 | 15 | 15 | 15 | 15 | 14 | 14 | 14 | 14 | 14 |
| Grenada | 17 | 17 | 17 | 16 | 16 | 15 | 15 | 15 | 15 | 15 | 14 | 14 | 14 | 14 | 14 | 14 | 14 |
| Guyana | 18 | 17 | 17 | 16 | 16 | 16 | 16 | 15 | 15 | 15 | 15 | 15 | 15 | 15 | 15 | 15 | 15 |
| Dominica | 19 | 18 | 18 | 17 | 17 | 16 | 16 | 16 | 16 | 16 | 15 | 15 | 15 | 15 | 14 | 14 | 14 |
| Haiti | 27 | 27 | 26 | 26 | 25 | 25 | 25 | 24 | 24 | 24 | 24 | 24 | 23 | 23 | 23 | 23 | 23 |
| **Central America** |  |  |  |  |  |  |  |  |  |  |  |  |  |  |  |  |  |
| Costa Rica | 13 | 12 | 12 | 12 | 12 | 11 | 11 | 11 | 11 | 11 | 11 | 11 | 11 | 11 | 11 | 11 | 11 |
| Panama | 15 | 16 | 16 | 15 | 15 | 15 | 15 | 15 | 15 | 15 | 15 | 15 | 14 | 14 | 14 | 13 | 13 |
| Belize | 19 | 18 | 18 | 17 | 17 | 16 | 16 | 15 | 15 | 15 | 14 | 14 | 14 | 14 | 14 | 14 | 13 |
| El Salvador | 19 | 19 | 18 | 17 | 17 | 16 | 16 | 16 | 16 | 16 | 15 | 15 | 15 | 15 | 15 | 15 | 15 |
| Mexico | 20 | 19 | 19 | 18 | 17 | 17 | 16 | 16 | 15 | 15 | 15 | 14 | 14 | 14 | 14 | 14 | 14 |
| Nicaragua | 23 | 22 | 21 | 21 | 20 | 19 | 18 | 17 | 16 | 15 | 15 | 14 | 13 | 13 | 13 | 13 | 13 |
| Honduras | 24 | 23 | 23 | 22 | 22 | 22 | 21 | 21 | 21 | 21 | 21 | 20 | 20 | 20 | 19 | 19 | 19 |
| Guatemala | 30 | 29 | 28 | 28 | 27 | 26 | 26 | 26 | 26 | 25 | 25 | 25 | 24 | 24 | 24 | 24 | 24 |
| **South America** |  |  |  |  |  |  |  |  |  |  |  |  |  |  |  |  |  |
| Chile | 10 | 10 | 9 | 9 | 8 | 8 | 8 | 8 | 7 | 7 | 7 | 7 | 7 | 7 | 7 | 7 | 7 |
| Argentina | 13 | 12 | 12 | 11 | 11 | 11 | 11 | 11 | 11 | 10 | 10 | 10 | 10 | 10 | 10 | 10 | 10 |
| Venezuela (Bolivarian Republic of) | 14 | 13 | 13 | 13 | 13 | 13 | 13 | 13 | 13 | 13 | 13 | 13 | 13 | 12 | 12 | 12 | 12 |
| Uruguay | 15 | 14 | 14 | 13 | 13 | 13 | 13 | 12 | 12 | 12 | 12 | 11 | 11 | 11 | 11 | 11 | 11 |
| Paraguay | 16 | 16 | 15 | 15 | 15 | 15 | 15 | 14 | 14 | 14 | 14 | 14 | 13 | 13 | 13 | 13 | 13 |
| Brazil | 16 | 16 | 15 | 14 | 14 | 13 | 13 | 13 | 12 | 12 | 12 | 12 | 11 | 11 | 11 | 11 | 11 |
| Suriname | 18 | 17 | 17 | 16 | 16 | 16 | 16 | 15 | 15 | 15 | 15 | 14 | 14 | 14 | 14 | 13 | 13 |
| Colombia | 18 | 18 | 18 | 17 | 17 | 17 | 17 | 17 | 16 | 16 | 16 | 15 | 15 | 14 | 14 | 13 | 13 |
| Peru | 23 | 22 | 22 | 21 | 21 | 21 | 20 | 20 | 20 | 20 | 19 | 19 | 18 | 18 | 17 | 17 | 16 |
| Ecuador | 23 | 23 | 22 | 22 | 21 | 21 | 21 | 20 | 20 | 20 | 19 | 19 | 19 | 18 | 18 | 18 | 18 |
| Bolivia | 24 | 23 | 23 | 22 | 22 | 21 | 21 | 21 | 21 | 20 | 20 | 20 | 20 | 19 | 19 | 19 | 19 |
