# Supplementary material for: Global Update and Trends of Hidden Hunger, 1995-2011: The Hidden Hunger Index
Source: PLoS One. 2015 Dec 16;10(12):e0143497. doi: 10.1371/journal.pone.0143497 (PMC4684416; doi:10.1371/journal.pone.0143497)
Supplement: S2 Table — (DOCX) [file pone.0143497.s002.docx]

**S2 Table: Comparison of the Hidden Hunger Index (HHI) estimates for 2009 (unweighted) using dietary zinc data as compared to using stunting prevalence as an estimate of the prevalence of zinc deficiency**

|  | | **Using stunting prevalence** | | **Using dietary zinc estimates** | |
| --- | --- | --- | --- | --- | --- |
| **Region** | | **Mean HHI (2009)** | **SD** | **Mean HHI**  **(2009)** | **SD** |
| **Africa** | | 40.4 | 5.0 | 37.3 | 4.3 |
|  | **West and Central Africa** | 39.1 | 4.4 | 35.8 | 3.5 |
|  | **East and Southern Africa** | 41.9 | 5.4 | 38.8 | 4.6 |
| **Americas** | | 25.7 | 2.6 | 24.9 | 3.2 |
|  | **Central America** | 28.0 | 3.0 | 27.8 | 3.1 |
|  | **South America** | 26.1 | 1.5 | 24.6 | 2.1 |
|  | **Caribbean** | 24.0 | 1.9 | 23.3 | 2.8 |
| **Middle East and North Africa** | | 28.0 | 5.8 | 27.9 | 4.0 |
|  | **Middle East** | 26.7 | 4.1 | 27.5 | 1.7 |
|  | **North Africa** | 31.1 | 8.3 | 28.5 | 6.5 |
| **Central and Eastern Europe** | | 27.8 | 3.5 | 25.8 | 3.3 |
| **South Asia** | | 37.9 | 5.8 | 34.2 | 3.9 |
| **East Asia and the Pacific** | | 33.1 | 4.3 | 29.5 | 3.7 |
| **Global** | | 33.0 | 7.6 | 30.7 | 6.5 |

SD, standard deviation of the country-level mean HHI across the countries in each region or sub-region
